# Health belief model for coronavirus infection risk determinants

**DOI:** 10.11606/s1518-8787.2020054002494

**Published:** 2020-04-28

**Authors:** Marcelo Fernandes Costa

**Affiliations:** I Departamento de Psicologia Experimental Instituto de Psicologia Universidade de São Paulo São PauloSP Brasil Departamento de Psicologia Experimental . Instituto de Psicologia . Universidade de São Paulo . São Paulo , SP , Brasil .; II Núcleo de Neurociências Aplicada Universidade de São Paulo São PauloSP Brasil Núcleo de Neurociências Aplicada . Universidade de São Paulo . São Paulo , SP , Brasil .

**Keywords:** Coronavirus Infections, prevention & control, Coronavirus Infections, psychology, Risk Reduction Behavior, Health Knowledge, Attitudes, Practice

## Abstract

**OBJECTIVE:**

To use the advantages of a ratio scale with verbal anchors in order to measure the risk perception in the novel coronavirus infection, which causes covid-19, in a health belief model-based questionnaire, as well as its validity and reproducibility.

**METHOD:**

We used the health belief model, which explores four dimensions: perceived susceptibility (five questions), perceived severity (five questions), perceived benefits (five questions), and perceived barriers (five questions). Additionally, we included a fifth dimension, called pro-health motivation (four questions). The questions composed an electronic questionnaire disseminated by social networks for an one-week period. Answers were quantitative values of subjective representations, obtained by a psychophysically constructed scale with verbal anchors ratio (CentiMax ^®^ ). Mean time for total filling was 12 minutes (standard deviation = 1.6).

**RESULTS:**

We obtained 277 complete responses to the form. One was excluded because it belonged to a participant under 18 years old. Reproducibility measures were significant for 22 of the 24 questions in our questionnaire (Cronbach’s α = 0.883). Convergent validity was attested by Spearman-Brown’s split half reliability coefficient (r = 0.882). Significant differences among groups were more intense in perceived susceptibility and severity dimensions, and less in perceived benefits and barriers.

**CONCLUSION:**

Our health belief model-based questionnaire using quantitative measures enabled the confirmation of popular beliefs about covid-19 infection risks. The advantage in our approach lays in the possibility of quickly, directly and quantitatively identifying individual belief profiles for each dimension in the questionnaire, serving as a great ally for communication processes and public health education.

## INTRODUCTION

In recent years, several viruses have drawn the medical and scientific community attention for presenting great risk to international public health. Among them are the coronaviruses, with great international projection due to the severe respiratory syndromes they cause, with middle east respiratory syndrome (MERS) and severe acute respiratory syndrome (SARS) being the best known ^1^ .

A recent outbreak of human infection by a novel coronavirus (2019-nCoV, now named SARS-CoV-2) has been reported in Wuhan City, Hubei Province, China. Until January 10, 2020, there were 41 reported cases for SARS-CoV-2 infection between December 8, 2019 and January 2, 2020 ^2^ . Due to the rapid virus spread through different Chinese cities, on February 13, 2020 the Chinese government announced 59,901 patients with a concordant diagnosis of pneumonia and 1,368 people killed by the novel coronavirus infection. Then the World Health Organization (WHO) officially named the 2019-nCoV infection coronavirus disease 2019 (covid-19) ^3 , 4^ .

As a result of the easy mobility across countries, covid-19 cases spread to other countries rapidly and intensely. This has led authorities from several countries to adopt non-pharmaceutical control measures to avoid transmission, such as social isolation. Some countries, such as Italy and Spain, have faced difficulties in population adhesion to the measure. Brazil has adopted similar strategies to control the virus transmission. Such populational difficulty in adhesion to behavioral controls may likewise occur in our country.

Therefore, understanding the determinants responsible for people’s resistance to protective measures against the virus spread is of great importance for the effectiveness of social isolation-based public policies, avoiding or reducing non-adherence to the proposed social controls. We expect the health believe model to help finding determinants for such behavior.

The health belief model is a tool developed to explain patient’s behavior in the face of an illness or the risk of falling ill ^5^ . It was developed in the 1950s and considers that positive factors increase pro-health behaviors while negative factors decrease or inhibit them. ^7^ Thus, to adopt a health care behavior and/or avoid risks for diseases, the patient must: (1) believe to be susceptible to the disease; (2) believe that the disease will negatively impact, at least moderately, their life; (3) believe that adopting certain behaviors is indeed beneficial to reduce their susceptibility or, if they already have it, its severity; (4) overlap important psychological barriers, key for a successful prevention or treatment ^8 , 10 , 11^ . Among other uses, this model has been successfully applied to assess diabetes severity ^12^ , analyze protective factors for bulimia ^6^ find determinants for oral health care ^13^ and study different cultures perception on dementias ^11^ .

Therefore, this study aims to use the expanded health belief model to map the profile of our population in view of the behavioral controls imposed by covid-19 in our country. To increase the ability to analyze the responses, we have used a psychophysical scaling methodology by ratio measurement, by a *Borg CR* ^®^ ratio scale with verbal anchorage (centiMax ^®^ ) ^14^ . This methodology showed to be highly efficient regarding ratio measurements acquisition as it facilitates quantitative responses for anchoring the corresponding verbal descriptors. Our experiments showed that the use of ratio scales with verbal anchors improves significantly the sensitivity and quantitative measurement of major depression scores ^19^ . Studies within the literature usually base the belief model on ordinal measurements, obtained through a Likert-type scale ^5 , 20^ . A considerable innovative advance in our study is the use of a quantitative ratio scale ^21^ . Thus, there is a higher amount of information obtained, enabling the use of quantitative and statistical tools of high predictive power, unlike ordinal scales, which restrict information to frequency levels with mode and association non-parametric analyses.

## METHODS

### Sample

The collection period was from March 17 to 24, 2020. The collected demographic data consisted of sex, age, schooling level, type of health system used, annual income, marital status, ethnicity or race, transportation system used to daily locomotion and chronic diseases.

Answers were obtained using a digital form disseminated in social networks (REDCap), characterizing a convenience sampling and snowball recruitment method. After explaining the experiment purpose, age was inquired. If the participant was under 18 years old, page was automatically redirected to the appreciation for the participation message. If they were older than 18, they could follow to the page containing the informed consent form. Once it was accepted, instructions were provided and testing began. Total time spent for questionnaire completion was 12 minutes (standard deviation [SD] = 1.6). The study follows the 1964 Declaration of Helsinki and its revised versions.

### Procedures

Participants were consulted about their beliefs and knowledge regarding covid-19 contamination. The questionnaire consisted of 24 questions. Perceived susceptibility, severity, benefits and barriers contained five questions each, besides four additional questions regarding behaviors and attitudes aimed at improving general health, which we call pro-health motivation.

Perceived susceptibility corresponds to knowledge and belief about coronavirus infection probability (e.g., “based on my overall health, my chance of catching coronavirus disease is...”). Perceived severity investigates the personal belief regarding individual suffering of the disease process and intensity of symptoms (e.g., “if I caught coronavirus disease, the chance of getting too impaired to do my daily activities would be...”). Perceived benefits concern the effectiveness of the behavioral mechanisms adopted to prevent the infection (e.g.: if I wear a mask, the chance to catch coronavirus disease by walking in the street or at work is...”). Perceived barriers approach the difficulties in respecting norms and instructions for protection and avoidance of coronavirus infection (e.g., “I think the possibility of using alternative transportation to come and go from my job instead of public transportation is...”). We expanded the original model by including the item pro-health motivation, which presents questions adopted for general health improvement (e.g., eating habits, exercise routine, etc.).

**Figure 1:**
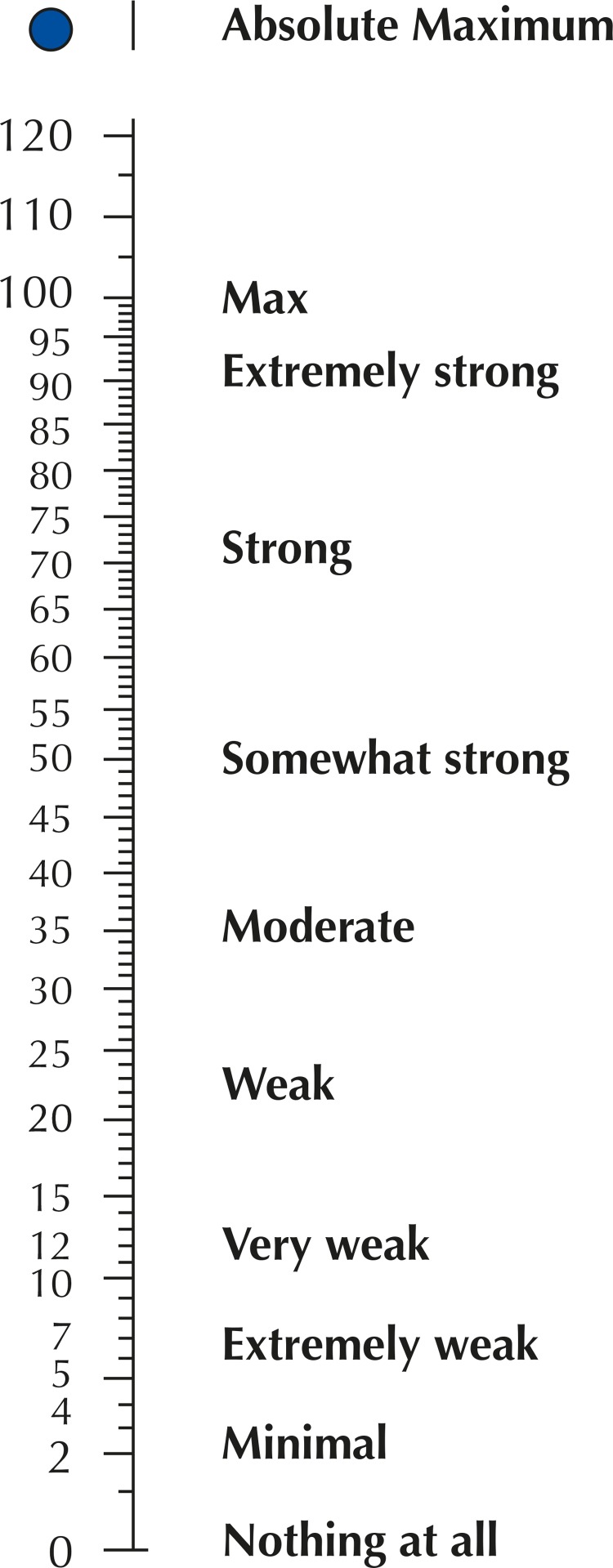
Illustration of the *Borg CR* ® (centiMax ® , CR100) ratio scale, in which anchored adjectives enable a quick access to the numerical region representing its intensity/magnitude perception (Borg & Borg, 2002; Borg et al., 2010). Scale and instructions can be obtained at www.borgperception.se. Authorized use.

The responses obtained were numerical values of a ratio scale with verbal anchors, derived from the centiMax scale ^14 , 24^ that represents perception ( Figure 1 ). This scale enables a direct quantitative measure of the participant perception degree on a psychophysical ratio scale ^22 , 25 , 26^ . The advantage of this quantitative method with verbal anchors is that qualitative descriptive anchors help participants to quickly locate the region of numeric scale values, in Cmax units, that represent their perception. Up from this point, a numerical value is chosen, always seeking the choice that indicates the most accurate representation.

Participants were instructed to choose the number that best represented the question-related perception, guided by the descriptors. Values could be integers or even contain decimals. It was crucial that the numerical information was as accurate as possible in representing participant’s perception or behavior.

### Data Analysis

The Statistica (version 10.2, Statsoft, Tulsa, USA) program was used for statistical analysis. An asymmetry test was performed to establish the necessary condition for parametric statistics, i.e. normality. Although centiMax ^®^ ratio scale has been successfully used to study different health conditions such as major depression ^19^ , fatigue and shortness of breath ^27^ , physical exertion ^15^ and chest pain ^28^ , it has not yet been applied in studies of purely psychological variables. Therefore, it is important to test some psychometric parameters. For reliability, we used split-half correlations with Spearman-Brown’s correction, Cronbach’s α for internal consistency and total items statistics to identify possible variables with low contribution to the questionnaire. We also calculated the standardized alpha, the reliability if we used standardized values (transformed into z score) for the items in cronbach’s α calculation. Repeated measures variance analysis was used to investigate the association between scale dimensions and sociodemographic characteristics, such as income level, type of health system and transportation system used to daily locomotion, through statistics F. Effect size was measured by h ^2^ . Spearman’s correlation was performed among scale values and demographic data on age, schooling level and income to attest to our measure convergence validity. To illustrate the possible added value of proportion data, belief profiles on coronavirus infection were built.

## RESULTS

Our study collected data from 277 participants. The responses of 276, aged between 18 and 76, of both sexes (women = 197) were used. The excluded response was given by an 11-year-old participant. Table 1 shows the summary of the demographic data of the sample.

**Table 1:** Demographic data of the sample (N = 276)

Participants’ characteristics	Percentage (n)
Age	40.3 (13.6) ^a^
Sex	
Men	29% (79)
Women	71% (197)
Ethnicity or race	
White	77% (213)
Mixed race	13% (37)
Black	2.3% (7)
Yellow (Asian)	7% (17)
Indigenous	0.7% (2)
Marital status	
Single	43% (119)
Married/Common-law marriage	48% (132)
Divorced/widowed	9% (25)
Schooling	
Graduate studies	42% (117)
College	33% (91)
Some College	18% (49)
Secondary education	5% (14)
Some secondary education	0.5% (1)
Some / primary education	1.5% (4)
No formal education	0
Monthly household income	
Over 10,001,00	28% (76)
Between R$ 5,001.00 and R$ 10,000.00	31% (85)
Between R$ 3,001.00 and R$ 5,000.00	21% (59)
Between R$ 1,001.00 and R$ 3,000.00	19% (52)
Less than 1,000.00	1% (3)
Locomotion transportation	
Public transportation	37% (101)
Private vehicles (taxi, apps and alike)	7% (16)
Personal vehicle	49% (135)
Walking	7% (16)
Chronic diseases	
Arterial hypertension	10% (27)
Diabetes mellitus	7% (18)
Immunodeficiency disorders	12% (33)
Respiratory disorders	6% (11)
None	65% (179)
Health system used	
Public	32% (88)
Private	68% (187)

^a^ Mean (standard deviation)

Table 2 shows the descriptive measures of the sums for the answers of each belief model dimension. Mean values are similar, ranging between 52.5 (SD = 31.6) for the pro-health motivation dimension and 26.6 (SD = 23.0) for perceived benefits.

**Table 2:** Mean, standard deviation and 95% confidence interval (95%CI) of belief model dimensions.

Dimension	Mean	Standard deviation	95%CI
Perceived susceptibility	39.5	29.8	37.9–41.1
Perceived severity	41.7	33.8	39.9–43.6
Perceived benefits	26.6	23.0	25.1–28.0
Perceived barriers	48.3	40.0	46.2–50.4
Pro-health motivation	52.5	31.6	50.6–54.4

### Questionnaire Analysis

We performed an analysis of reproducibility for the questions of each belief model dimension. Initial values showed a very good cronbach’s α (αC = 0.817) as well as standardized α (αP = 0.821), and a low correlation among the items (r = 0.168), suggesting a great independence of the questions ( Table 3 ). However, for question 3 of perceived benefits and question 1 of perceived barriers, item-total correlation values were very low (r = 0.06 and r = 0.08, respectively), suggesting they interfere negatively in our scale.

**Table 3:** Mean, correlation and Cronbach’s α values per item before and after BeP3 and BaP1 removal.

Questions	Mean	Standard deviation	Item-total correlation	α		Mean	Standard deviation	Item-total correlation	α
	Initial form					Final form (no BeP3 and BaP1)			
SuP1	950.2	301.9	0.53	0.78		871.3	287.9	0.57	0.81
SuP2	957.6	301.3	0.47	0.78		878.7	287.3	0.51	0.81
SuP3	959.8	304.6	0.36	0.79		880.9	291.0	0.39	0.82
SuP4	965.4	304.1	0.52	0.78		886.8	290.9	0.54	0.81
SuP5	954.5	307.8	0.37	0.79		876.0	295.1	0.36	0.82
SeP1	950.5	303.7	0.38	0.79		872.2	290.8	0.39	0.82
SeP2	963.3	302.3	0.54	0.78		884.9	289.5	0.54	0.81
SeP3	951.5	301.3	0.49	0.78		873.2	288.6	0.49	0.81
SeP4	956.1	299.8	0.58	0.78		877.5	287.0	0.59	0.81
SeP5	956.2	308.3	0.37	0.79		877.4	295.2	0.38	0.82
BeP1	985.1	313.8	0.34	0.79		906.3	300.9	0.36	0.82
BeP2	974.5	309.7	0.32	0.79		895.8	296.9	0.33	0.82
BeP3	983.4	316.3	0.06	0.80		-	-	-	-
BeP4	962.6	306.8	0.38	0.79		884.2	294.2	0.37	0.82
BeP5	945.6	304.1	0.42	0.78		867.0	292.2	0.40	0.81
BaP1	933.3	307.7	0.08	0.82		-	-	-	-
BaP2	977.0	313.2	0.11	0.80		897.9	299.4	0.14	0.83
BaP3	948.2	306.0	0.22	0.80		869.6	293.5	0.21	0.83
BaP4	931.6	305.6	0.31	0.79		852.7	292.8	0.31	0.82
BaP5	949.1	311.7	0.15	0.80		870.4	300.1	0.11	0.83
MoS1	928.2	306.5	0.38	0.79		849.2	293.9	0.37	0.82
MoS2	951.3	305.3	0.39	0.79		872.6	292.4	0.40	0.81
MoS3	948.7	304.8	0.43	0.78		870.3	291.9	0.44	0.81
MoS4	948.2	303.9	0.37	0.79		869.8	290.6	0.39	0.82

PSU: perceived susceptibility; PSE: perceived severity; PBE: perceived benefits; PBA: perceived barriers; PHM: pro-health motivation.

Note: The numbers represent the question numbers for each model dimension. Values are in units of the centiMax scale ^®^ .

After the removal of these two questions, our scale showed a better cronbach’s α than the previous one (αC = 0.883), exceeding the 0.800 value. This allows to presumpt the high efficiency of the scale, since it represents 80% of the population expected variability. Likewise, standardized α presented a slight improvement (αP = 0.834), and the low correlation among items was maintained (r = 0.179), certifying an excellent internal consistency. Subsequent analyses were performed without the presence of these two questions.

The scale reproducibility was estimated by Spearman-Brown’s split half coefficient. The values found suggest a high correlation (r = 0.882), confirming a high convergent validity for our scale.

### Comparison between Groups

The analysis of variance of the grouped scores of the items of each belief model dimension showed differences for perceived susceptibility regarding: type of transportation, in which urban transportation presented higher mean values (44.0) than personal vehicle (36.1) and walking (30.2) (F = 5.21; p = 0.003; h ^2^ = 0.014); household income, in which participants with income lower than R$ 1,000.00 presented mean values (16.4) significantly lower than all other income groups (F = 3.44; p = 0.008; h ^2^ = 0.009); and different severe illnesses, in which participants with autoimmune diseases (83.1) and diseases affecting the immune system (42.8) presented higher values than other diseases and those without severe illness (F = 3.13; p = 0.008; h ^2^ = 0.022).

For perceived severity, schooling level showed a significant difference (F = 2.79; p = 0.016; h ^2^ = 0.012), in which participants with complete secondary education (47.9) and incomplete (72.3) presented higher values than those with higher schooling level. Within the group with severe illness, participants with systemic arterial hypertension (47.9) and diseases affecting the immune system (47.8) reported higher values, whereas those with autoimmune diseases had lower values (22.5) than other groups (F = 10.79; p < 0.001; h ^2^ = 0.022).

Perceived benefits dimension was significant for different participants regarding the health system used (F = 4.32; p = 0.037; h ^2^ = 0.007), as participants who used private health systems (21.5) presented higher values than those who used the public system (18.2).

Perceived barriers were the last dimension to present significant differences among participants (F = 3.79; p = 0.004; h ^2^ = 0.014), in which public transportation users presented lower values than others groups. Significant differences regarding participants’ sex were not found, nor were significant correlations between age and scale responses.

### Individual Profile

**Figure 2:**
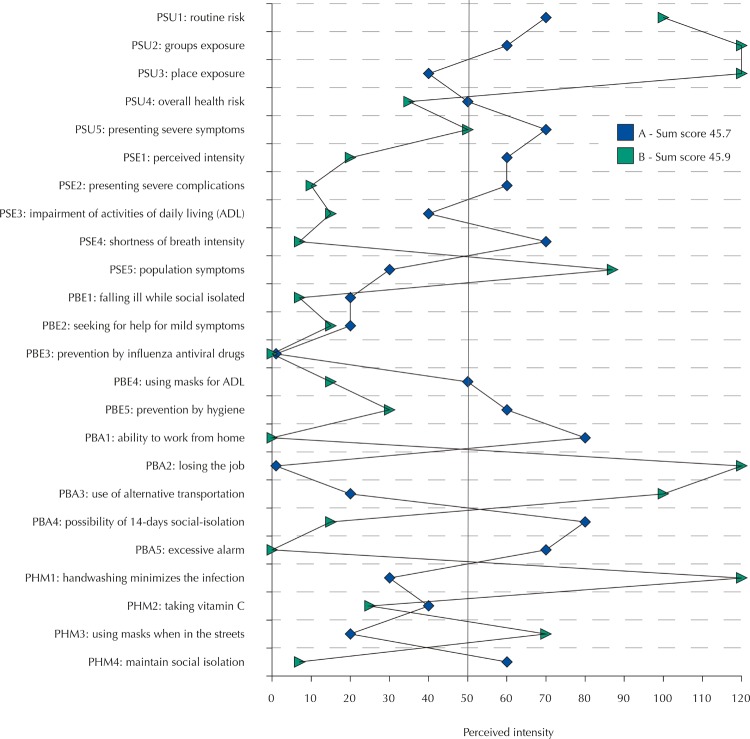
Representation profile on the belief of coronavirus infection (causing covid-19) for classification in centiMax scale ® units (Cmax) of the intensity of risk perception for two people with very similar sum scores (A = 45.7 and B = 45.9 units; risk chance self-declared as high). Vertical line at 50 units indicates a “high” infection risk perception on the scale (see Figure 1). PSU: perceived susceptibility; PSE: perceived severity; PBE: perceived benefits; PBA: perceived barriers; PHM: pro-health motivation. The numbers represent the question numbers for each model dimension. Values are in units of the centiMax scale ^®^ .

CentiMax scale ^®^ enables a directly and quickly graphical analysis of each participant. Figure 2 shows the profile of two participants who present the sum values of the scale items practically equal (participant A – 45.9; participant B – 45.7). However, different beliefs regarding covid-19 can be clearly identified. The vertical line in the value of 50 scale units represents the high perception of the item in question. From an application perspective, Figure 2 shows how our questionnaire associated with ratio scale use provides a quantitative and refined gradation, enabling the participant to express their respective perceptions and intensities with a higher level of detail when compared with other ordinal or nominal scales.

## DISCUSSION

Our study successfully fulfills our objective of quantitatively mapping risk behaviors perception in view of the coronavirus infection risk through the use of the health belief model associated with a verbal anchors psychophysical scale, enabling a detailed representation of individual perceptions. This approach has proved to be very efficient, by identifying in participants with the same value of risk perception very different profiles within the variables.

The results identified that some factors are significantly important to understand risk perception. The type of transportation used to get around on a daily basis significantly affects risk perception. Public transportation users perceive a greater contamination susceptibility than personal vehicle users and those who do their activities by foot. The same greater susceptibility perception was found in people with very low income and individuals with autoimmune diseases and diseases that affect the immune system (allergies and rheumatism, for example).

These findings are important sources of information for adopting public policies that seek the widest possible scope. The perception that using public transportation results in a higher covid-19 contraction risk than private and personal transportation is positive. However, the use of collective private vehicles, such as transportation apps, presented a great variability and, thus, did not differ from any of the groups. Therefore, we understand this is an interesting target audience to provide more information regarding contagion risks. Low income was also an important factor and should be seen in a complex way, as it is associated with reduced information quantity and quality, housing conditions that favor contamination and difficulty in interrupting daily activities due to economic reasons.

A fact that drew our attention was that people with diseases associated with a higher covid-19 contraction risk, such as diabetes mellitus and systemic arterial hypertension, ^1 , 3^ do not present a significant different susceptibility perception to contamination risk in comparison with the group of people without self-reported chronic diseases. A possible explanation is that these patients are mostly asymptomatic and remain with a stable and clinically controlled disease, behaving as individuals without chronic diseases. Such result allows us to develop information dissemination policies emphasizing or even particularly targeting this risk group.

Regarding intensity of symptoms and characteristics of disease progression, participants with the lower schooling levels in our sample (some and secondary education) showed greater concern with possible symptoms, because their perceived severity representations were higher than in other schooling levels. The perception of greater symptom severity may lead these group to seek health services earlier. This problem is currently at stake due to the risk of unnecessary covid-19 exposure, as well as other serious diseases, by visiting health institution without real need.

Another curious result for severity perception was obtained regarding chronic diseases. Participants with systemic arterial hypertension and diseases affecting the immune system registered higher susceptibility than other groups. However, participants with autoimmune diseases reported significantly lower values than others, suggesting that precautions taken for the treatment and care of their chronic diseases are being positively perceived as protective factors to the exposure. The type of health system used had an impact on benefits perception regarding the access and treatment in case of covid-19. Public system users were less benefited in comparison with participants who use private health systems.

Results obtained through individual profiles enable highly informative and direct quantitative analyses. For example, if we look at question 1 of the health motivation item in Figure 2 , we notice that participant A believes 30 units that the behavior of washing their hands prevents the infection by the virus. Participant B believes 120 units that this behavior is beneficial. For being a ratio scale, we can directly state that participant B believes four times more than participant A in the effectiveness of washing hands as a healthy behavior that should be encouraged or increased. When comparing question 2 of the perceived severity item, participant A believes twice as much in the chance of presenting severe symptoms and complications as participant B. If a Likert-type scale of 5 points were used, in the first case, the result would have been too high (or value 5, if a numerical score) for participant B and low (value 2) for participant A. In the second case, participant A would present a moderate result (or value 3) and participant B a low result (value 2). In this example, there is a clear difference between the use of a ratio scale and an ordinal scale, whose sensitivity and resolution impair the actual identification of participants’ representations.

It is also significantly important to discuss the methodology used in this study. The health belief model was developed over 60 years ago ^8 , 9^ and is applied in several areas of health – such as ophthalmologist education ^29^ , study of behavioral aspects in eating behavior psychiatry ^6^ , use and abuse of illicit injectable drugs ^30^ , clinics for diabetes mellitus and endocrine ^12^ , among others. However, the customary use of this model includes open or semi-structured questionnaires, using Likert-type scales to perform psychological dimension measurements. Our study is a clear advance in the use of this model, as it applies a ratio scale with verbal anchors, allowing ratio measures and, therefore, presenting a high-potential information detailing in a quantitative manner. This methodology is noteworthy because even a good questionnaire may have its information gathering potential greatly impaired if an inadequate or low information capacity metric is used. S.S. Stevens’ work on psychological measurement and psychophysical scaling ^21 , 23^ expanded our understandings on metric possibilities applied into the psychological universe. Likert-type scales are ordinal and, hence, positional. Their descriptors solely indicate orders and are unable to designate the distance among them. Therefore, ordinal scales use nonparametric statistical measurement such as mode, frequency, association and categorical correlation. Estimating medians on Likert-type scales, although the order is numerically represented, is a fundamental measurement error. On the other hand, measurements within ratio universes, for presenting an absolute origin of mathematical continuums, enable higher-order statistics. Our study, therefore, presents a high information quality and objective metric.

Our group is already experienced in constructing psychophysical measurement and scaling of interval and ratio orders for studies of various psychological *continua* , such as symptoms profile in patients with major depression ^19^ , sexual attitudes ^31^ and color concept ^32^ . We encourage the use of these psychophysical scaling models, especially verbal anchors scales, because they are easy to apply and to understand the task to be performed. In addition, they are associated with quantitative measurements of high capacity for information, intervals and ratio. Such factors foster a great potential for more detailed and quantitative studies in public health, which, like psychology, commonly faces complex characteristics of human behavior regarding prevention issues as well as health and illness risks.

Our study presents some evident limitations, which may have impacted some of the results. Our sample presents a thickening in moderate- and high-income populations, which may affect the sample representativeness. Therefore, subsequent studies should seek to correct this social heterogeneity within the sample.

In conclusion, our health belief model-based questionnaire associated with a ratio scale with verbal anchors is an important instrument to understand the population perception on coronavirus infection risks in a quantitative manner, much more detailed and informative than the commonly applied questionnaires models and psychological metrics, such as Likert-type ordinal scales. The graphical analysis grants a quick access to the individual profile, enabling the development of information strategies and more individualized approaches, which will certainly have a greater impact on communication efficiency.
