# Supplementary material for: Health belief model for coronavirus infection risk determinants
Source: Rev Saude Publica. 2020 Apr 28;54:47. doi: 10.11606/s1518-8787.2020054002494 (PMC7190095; doi:10.11606/s1518-8787.2020054002494)
Supplement: Translation

# Translation: Modelo de crença em saúde para determinantes de risco para contaminação por coronavírus

**Authors:** Marcelo Fernandes Costa

**Affiliations:** I Brasil Departamento de Psicologia Experimental. Instituto de Psicologia. Universidade de São Paulo. São Paulo, SP, Brasil .; II Brasil Núcleo de Neurociências Aplicada. Universidade de São Paulo. São Paulo, SP, Brasil .

**Keywords:** Infecções por Coronavirus, prevenção & controle, Infecções por Coronavirus, psicologia, Comportamento de Redução do Risco, Conhecimentos, Atitudes e Prática em Saúde

## Abstract

**OBJETIVO:**

Neste estudo buscamos utilizar as vantagens de uma escala de razão por ancoragem verbal para medidas da percepção de risco de contágio pelo novo coronavírus, causador da covid-19, em um questionário baseado no modelo de crença em saúde, assim como avaliar sua validade e reprodutibilidade.

**MÉTODO:**

Utilizamos o modelo de crença em saúde, o qual explora quatro dimensões: percepção individual de susceptibilidade percebida (cinco questões), severidade percebida (cinco questões), benefícios percebidos (cinco questões) e barreiras percebidas (cinco questões). Adicionalmente, incluímos uma quinta dimensão, a qual denominamos motivação pró-saúde (quatro questões). As questões definiram um questionário eletrônico que foi divulgado por redes sociais pelo período de uma semana. As respostas foram valores quantitativos de representações subjetivas, obtidas por meio de uma escala psicofísica de razão com ancoragem verbal (CentiMax ^®^ ). O tempo médio total de preenchimento foi de 12 minutos (desvio-padrão = 1,6).

**RESULTADOS:**

Obtivemos 277 respostas completas ao formulário. Uma foi excluída por se tratar de participante com menos de 18 anos de idade. Medidas de reprodutibilidade foram significantes para 22 das 24 questões de nosso questionário (α de Cronbach = 0,883). A validade convergente foi atestada pelo coeficiente de correlação de Spearman-Brown *split half* (r = 0,882). Diferenças significantes entre grupos foram encontradas mais intensamente nas dimensões susceptibilidade percebida e severidade percebida, e menos intensamente para benefícios percebidos e barreiras percebidas.

**CONCLUSÃO:**

Nosso questionário baseado no modelo de crença em saúde utilizando medidas quantitativas permitiu evidenciar as crenças populares sobre os riscos de contágio por covid-19. A vantagem de nossa abordagem é a possibilidade de se identificar os perfis de crença individuais para cada dimensão do questionário de forma rápida, direta e quantitativa, podendo ser uma grande aliada em processos de comunicação e educação em saúde pública.

## INTRODUÇÃO

Nos últimos anos, vários vírus têm chamado atenção da comunidade médica e científica por serem de grande risco para a saúde pública internacional. Entre eles estão os coronavírus, que tiveram grande projeção internacional pelo fato de causarem síndromes respiratórias graves, sendo as mais conhecidas a síndrome respiratória do Oriente Médio (MERS) e a síndrome respiratória aguda severa (SARS) ^1^ .

Um recente surto de infecção humana por um novo coronavírus (2019-nCoV, atualmente denominado SARS-CoV-2) foi relatado na cidade de Wuhan, província de Hubei, China. Até 10 de janeiro de 2020, foram relatados 41 casos de contágio por SARS-CoV-2 entre 8 de dezembro de 2019 e 2 de janeiro de 2020 ^2^ . Pelo rápido espalhamento do vírus por diferentes cidades chinesas, no dia 13 de fevereiro de 2020, o governo chinês anunciou 59.901 pacientes com diagnóstico confirmado de pneumonia e 1.368 pessoas mortas pela infecção do novo coronavírus, e então a Organização Mundial da Saúde (OMS) nomeou oficialmente a infecção por 2019-nCoV como doença por coronavírus 2019 (covid-19) ^3,4^ .

Devido às facilidades de transporte entre países presentes na vida moderna, o avanço dos casos de covid-19 para outros países foi rápido e intenso. Isso tem levado as autoridades desses países a adotarem medidas de controle de transmissão não medicamentoso, como, por exemplo, o isolamento social. Alguns países, como a Itália e a Espanha, encontraram dificuldade para que a população aderisse ao tratamento. O Brasil tem adotado estratégias semelhantes de controle da transmissão do vírus. Essa dificuldade que a população demonstrou para aderir aos controles comportamentais pode, também, se repetir em nosso país.

Entender os determinantes pelos quais as pessoas podem apresentar relativa resistência às medidas protetivas contra a disseminação do vírus é, claramente, de grande importância para que as políticas públicas baseadas no isolamento social possam ter a eficácia desejada, evitando ou reduzindo a não aderência aos controles sociais propostos. Acreditamos que o modelo de crença em saúde possa auxiliar em encontrar os determinantes para tal comportamento.

O modelo de crença em saúde é uma ferramenta desenvolvida para explicar o comportamento do paciente diante de sua doença ou risco de adoecer ^5–7^ . Ele surgiu na década de 1950 e considera que fatores positivos aumentam comportamentos pró-saúde enquanto fatores negativos os diminuem ou inibem ^7–9^ . Assim, para adotar comportamento de cuidado com a saúde e/ou de evitação de risco para doenças, o paciente deve: (1) acreditar que é suscetível à doença; (2) acreditar que a doença gerará impacto negativo, ao menos moderado, em sua vida; (3) acreditar que adotar determinados comportamentos é, de fato, benéfico para reduzir sua susceptibilidade ou a severidade da doença, caso já a tenha; (4) sobrepor importantes barreiras psicológicas, o que é fundamental para o sucesso da prevenção ou do tratamento ^8,10,11^ . Esse modelo tem sido aplicado com sucesso para avaliar a severidade da diabetes ^12^ , analisar os fatores de prevenção para a bulimia ^6^ , encontrar os determinantes de cuidados em saúde bucal ^13^ e estudar a percepção que diferentes culturas têm sobre as demências ^11^ , entre outros usos.

Neste trabalho buscamos, portanto, o uso do modelo de crença em saúde ampliado para mapear o perfil da nossa população diante das necessidades de modificação comportamentais impostas pela chegada do coronavírus em nosso país. Para aumentar a capacidade de análise das respostas obtidas, utilizamos uma metodologia de escalonamento psicofísico por medida de razão, utilizando uma escala de razão com ancoragem verbal *Borg CR scale* ^®^ (centiMax ^®^ ) ^14–18^ . Essa metodologia tem se mostrado altamente eficiente na obtenção de medidas de razão por facilitar as respostas quantitativas pela ancoragem de descritores verbais correspondentes. Nossas experiências mostraram que o uso de escalas de razão com ancoragem verbal tem melhorado significativamente a sensibilidade e a medida quantitativa de escores de depressão maior ^19^ . Os trabalhos na literatura habitualmente utilizam o modelo de crença baseado em medidas ordinais, obtidas por meio de escala do tipo Likert ^5,20^ . Um avanço inovador nítido em nosso trabalho é o uso de uma escala quantitativa de razão ^21–23^ . Dessa forma, a quantidade de informação obtida é maior e permite o uso de ferramentas quantitativas e estatísticas de alto poder de predição, diferentemente das escalas ordinais, que restringem a informação a níveis de frequência com análises não paramétricas de moda e associação.

## MÉTODOS

### Amostra

O período de coleta foi de 17 a 24 de março de 2020. Os dados demográficos coletados consistiram em sexo, idade, nível de escolaridade, tipo de sistema de saúde utilizado, renda anual, estado civil, etnia ou raça, sistema de transporte utilizado para se locomover diariamente e doenças crônicas.

As respostas foram obtidas por meio de formulário digital disseminado em redes sociais (REDCap), caracterizando um modelo de amostragem por conveniência e modelo de recrutamento tipo *snowball* (bola de neve). Após a explicação do propósito do experimento, a idade era perguntada. Caso o participante tivesse menos de 18 anos, a página automaticamente era direcionada para os agradecimentos pela participação. Caso a idade fosse maior de 18 anos, seguia-se à página contendo o termo de consentimento livre e esclarecido (TCLE), após o aceite as instruções eram oferecidas e começava-se a testagem. Caso o participante não concordasse com o TCLE, a página automaticamente era direcionada para os agradecimentos pela participação. O tempo total dispendido para o preenchimento do questionário foi de 12 minutos (desvio-padrão [DP] = 1,6). O estudo segue os princípios da Declaração de Helsinki de 1964 e de suas versões revisadas.

### Procedimentos

Os participantes foram consultados sobre suas crenças e conhecimentos a respeito da infecção por covid-19. O questionário era composto por 24 questões, sendo cinco delas relacionadas à susceptibilidade percebida, cinco à severidade percebida, cinco aos benefícios percebidos e cinco às barreiras percebidas, além de quatro questões adicionais sobre comportamentos e atitudes que visam melhorar a saúde geral, as quais denominamos de motivação pró-saúde.

A susceptibilidade percebida corresponde ao conhecimento e crença sobre a possibilidade de pegar coronavírus (por exemplo, “com base na minha saúde geral, minha chance de pegar coronavírus é...”). A severidade percebida investiga a crença pessoal a respeito de como o indivíduo sofreria o processo da doença e a intensidade dos sintomas (ex.: “se eu pegasse coronavírus, a chance de ficar muito comprometido para fazer minhas atividades diárias seria...”). A dimensão de benefícios percebidos questiona a efetividade dos mecanismos comportamentais adotados para evitar a infecção (ex.: “se eu usar máscara, a chance de pegar coronavírus andando na rua ou no trabalho é...”), enquanto a de barreiras percebidas busca entender as dificuldades de respeitar as normas e instruções de proteção e evitação do contágio por coronavírus (ex.: “acho que a possibilidade de utilizar transporte alternativo para ir e vir do meu trabalho em vez de transporte público é...”). Ampliamos o modelo original pela inclusão em do item motivação pró-saúde, no qual apresentamos questões que são adotadas para melhora da saúde geral (por exemplo, tipo de alimentação, rotina de exercícios etc.).

As respostas obtidas foram valores numéricos de uma escala de razão com ancoragem verbal, derivada da escala centiMax ^® 14,24^ que representa sua percepção (Figura 1). Essa escala permite uma medida quantitativa direta do grau percebido pelo participante em uma escala psicofísica de razão ^22,25,26^ . A vantagem desse método quantitativo com ancoragem verbal é que as âncoras descritoras qualitativas auxiliam o participante a localizar rapidamente a região de valores da escala numérica, em unidades Cmax, que representam sua percepção. A partir desse ponto, um valor numérico é escolhido, buscando sempre a escolha que indique a representação mais precisa.

**Figura 1:** Ilustração da escala de razão *Borg CR scale* ® (centiMax ® , CR100), na qual os adjetivos ancorados permitem um rápido acesso à região numérica que representa sua percepção de intensidade/magnitude (Borg & Borg, 2002; Borg et al., 2010). A escala e as instruções podem ser obtidas em www.borgperception.se. Uso permitido.

Os participantes foram instruídos a escolher o número que melhor representasse a percepção relacionada à pergunta realizada, guiados pelos descritores. Os valores poderiam ser inteiros ou mesmo conter decimais. O importante é que a informação numérica fosse a que ele ou ela julgasse a mais precisa possível na representação de sua percepção ou comportamento.

### Análise dos Dados

O programa *Statistica* (versão 10.2, Statsoft, Tulsa, EUA) foi utilizado para análise estatística. Para estabelecer uma condição necessária para a estatística paramétrica, ou seja, normalidade, foi realizado um teste de assimetria. Embora a escala de razão centiMax ^®^ tenha sido usada com sucesso para estudar diferentes condições de saúde como depressão maior ^19^ , fadiga e falta de ar ^27^ , esforço físico ^15^ e dor no peito ^28^ , não foi aplicada em estudos de variáveis puramente psicológicas. Dessa forma, é importante testar alguns parâmetros psicométricos. Para confiabilidade, foram utilizadas correlações de meio a meio ( *split-half correlation* ) com correção de Spearman-Brown, o α de Cronbach para consistência interna e estatística total de itens para identificar possíveis variáveis com baixa contribuição para o questionário. Calculamos também o alfa padronizado, que é a confiabilidade se usássemos os valores padronizados (transformados em escore z) para os itens no cálculo do α de Cronbach. A análise de variância de medidas repetidas foi utilizada para investigar a associação das dimensões da escala com as características sociodemográficas como nível de renda, tipo de serviço de saúde e sistema de transporte utilizado para se locomover diariamente por meio da estatística F. O tamanho do efeito foi medido por η ^2^ . A correlação de Spearman foi realizada entre os valores da escala com dados demográficos de idade, escolaridade e renda para atestar a validade de convergência de nossa medida. Para ilustrar o possível valor agregado dos dados da proporção, foram construídos perfis de crenças sobre a contaminação por coronavírus.

## RESULTADOS

Nosso estudo coletou dados de 277 participantes e foram utilizadas as respostas de 276, com idade variando entre 18 e 76 anos de idade, de ambos os sexos (mulheres = 197). O resultado excluído tratava-se de resposta dada por participante com 11 anos de idade. O resumo dos dados demográficos da amostra está apresentado na Tabela 1.

**Tabela 1:** Dados demográficos da amostra (N = 276).

**Característica dos participantes**	**Porcentagem (n)**
Idade	40,3 (13,6)*
Sexo	
Masculino	29% (79)
Feminino	71% (197)
Raça ou cor	
Branca	77% (213)
Parda	13% (37)
Preta	2,3% (7)
Amarela	7% (17)
Indígena	0,7% (2)
Estado civil	
Solteiro(a)	43% (119)
Casado(a)/união estável	48% (132)
Divorciado(a)/viúvo(a)	9% (25)
Escolaridade	
Pós-graduação	42% (117)
Superior completo	33% (91)
Superior incompleto	18% (49)
Secundário completo	5% (14)
Secundário incompleto	0,5% (1)
Primário completo/incompleto	1,5% (4)
Sem instrução formal	0
Renda familiar mensal	
Maior que R$ 10.001,00	28% (76)
Entre R$ 5.001,00 e R$ 10.000,00	31% (85)
Entre R$ 3.001,00 e R$ 5.000,00	21% (59)
Entre R$ 1.001,00 e R$ 3.000,00	19% (52)
Menor que R$ 1.000,00	1% (3)
Transporte utilizado para deslocamento	
Transporte público	37% (101)
Veículos particulares (táxi, aplicativos e similares)	7% (16)
Veículo próprio	49% (135)
A pé	7% (16)
Doenças crônicas	
Hipertensão arterial	10% (27)
Diabetes mellitus	7% (18)
Problemas imunológicos	12% (33)
Problemas respiratórios	6% (11)
Nenhum	65% (179)
Sistema de saúde utilizado	
Público	32% (88)
Privado	68% (187)

* Média (desvio-padrão)

As medidas descritivas das somas para as respostas de cada dimensão do modelo de crença estão apresentadas na Tabela 2. Os valores médios são semelhantes, variando entre 52,5 (DP = 31,6) para a dimensão motivação pró-saúde e 26,6 (DP = 23,0) para benefícios percebidos.

**Tabela 2:** Média, desvio-padrão e intervalo de confiança de 95% (IC95%) das dimensões do modelo de crença.

**Dimensão**	**Média**	**Desvio-padrão**	**IC95%**
Susceptibilidade percebida	39,5	29,8	37,9–41,1
Severidade percebida	41,7	33,8	39,9–43,6
Benefícios percebidos	26,6	23,0	25,1–28,0
Barreiras percebidas	48,3	40,0	46,2–50,4
Motivação pró-saúde	52,5	31,6	50,6–54,4

SuP: susceptibilidade percebida; SeP: severidade percebida; BeP: benefícios percebidos; BaP: barreiras percebidas; MoS: motivação pró-saúde. Os números representam os números das perguntas de cada dimensão do modelo. Os valores estão em unidades da escala centiMax ^®^ .

### Análise do Questionário

Realizamos uma análise de reprodutibilidade para as questões de cada uma das dimensões do modelo de crenças. Os valores iniciais mostraram um α de Cronbach muito bom (αC = 0,817), assim como o α padronizado (αP = 0,821) e uma baixa correlação entre itens (r = 0,168), sugerindo grande independência das questões (Tabela 3). No entanto, para a questão 3 da dimensão benefícios percebidos e para a questão 1 das barreiras percebidas, os valores de correlação item-total foram muito baixos (r = 0,06 e r = 0,08, respectivamente), sugerindo que elas atuam como interferências negativas em nossa escala.

**Tabela 3:** Valores de média, correlação e α de Cronbach por item antes e depois da retirada de BeP3 e BaP1.

**Questões**	**Média**	**Desvio-padrão**	**Correlação item-total**	**α**		**Média**	**Desvio-padrão**	**Correlação item-total**	**α**
	Formulário inicial					Formulário final (sem BeP3 e BaP1)			
SuP1	950,2	301,9	0,53	0,78		871,3	287,9	0,57	0,81
SuP2	957,6	301,3	0,47	0,78		878,7	287,3	0,51	0,81
SuP3	959,8	304,6	0,36	0,79		880,9	291,0	0,39	0,82
SuP4	965,4	304,1	0,52	0,78		886,8	290,9	0,54	0,81
SuP5	954,5	307,8	0,37	0,79		876,0	295,1	0,36	0,82
SeP1	950,5	303,7	0,38	0,79		872,2	290,8	0,39	0,82
SeP2	963,3	302,3	0,54	0,78		884,9	289,5	0,54	0,81
SeP3	951,5	301,3	0,49	0,78		873,2	288,6	0,49	0,81
SeP4	956,1	299,8	0,58	0,78		877,5	287,0	0,59	0,81
SeP5	956,2	308,3	0,37	0,79		877,4	295,2	0,38	0,82
BeP1	985,1	313,8	0,34	0,79		906,3	300,9	0,36	0,82
BeP2	974,5	309,7	0,32	0,79		895,8	296,9	0,33	0,82
BeP3	983,4	316,3	0,06	0,80		-	-	-	-
BeP4	962,6	306,8	0,38	0,79		884,2	294,2	0,37	0,82
BeP5	945,6	304,1	0,42	0,78		867,0	292,2	0,40	0,81
BaP1	933,3	307,7	0,08	0,82		-	-	-	-
BaP2	977,0	313,2	0,11	0,80		897,9	299,4	0,14	0,83
BaP3	948,2	306,0	0,22	0,80		869,6	293,5	0,21	0,83
BaP4	931,6	305,6	0,31	0,79		852,7	292,8	0,31	0,82
BaP5	949,1	311,7	0,15	0,80		870,4	300,1	0,11	0,83
MoS1	928,2	306,5	0,38	0,79		849,2	293,9	0,37	0,82
MoS2	951,3	305,3	0,39	0,79		872,6	292,4	0,40	0,81
MoS3	948,7	304,8	0,43	0,78		870,3	291,9	0,44	0,81
MoS4	948,2	303,9	0,37	0,79		869,8	290,6	0,39	0,82

SuP: susceptibilidade percebida; SeP: severidade percebida; BeP: benefícios percebidos; BaP: barreiras percebidas; MoS: motivação pró-saúde.

Nota: Os números representam os números das perguntas de cada dimensão do modelo. Os valores estão em unidades da escala centiMax ^®^ .

Após a retirada dessas duas questões, nossa escala mostrou um α de Cronbach melhor que o anterior (αC = 0,883), inclusive ultrapassando o valor de 0,800, que permite assumir uma alta eficiência da escala, uma vez que representa 80% da variabilidade esperada na população. Da mesma forma, o α padronizado teve uma leve melhora (αP = 0,834) e mantivemos uma baixa correlação entre itens (r = 0,179), atestando uma ótima consistência interna. As análises posteriores foram realizadas sem a presença dessas duas questões.

Nós estimamos a reprodutibilidade da escala pelo coeficiente de Spearman-Brown *split half* . Os valores encontrados sugerem uma correlação alta (r = 0,882), confirmando uma alta validade convergente para nossa escala.

### Comparação entre Grupos

A análise de variância dos escores agrupados dos itens pertencentes a cada dimensão do modelo de crenças mostrou haver diferenças para susceptibilidade percebida por tipo de transporte, com o transporte urbano apresentando maiores valores médios (44,0) que veículo próprio (36,1) e a pé (30,2) (F = 5,21; p = 0,003; η ^2^ = 0,014); por renda familiar, com participantes de renda menor do que R$ 1.000,00 apresentando valores médios (16,4) significativamente menores do que todos os outros grupos de renda (F = 3,44; p = 0,008; η ^2^ = 0,009); e para diferentes doenças de risco, uma vez que participantes com doenças autoimunes (83,1) e doenças que afetam o sistema imunológico (42,8) apresentaram valores maiores do que outras doenças e do que o grupo sem doença de risco (F = 3,13; p = 0,008; η ^2^ = 0,022).

Para a dimensão severidade percebida, a escolaridade apresentou diferença significante (F = 2,79; p = 0,016; η ^2^ = 0,012), com participantes com formação secundária completa (47,9) e incompleta (72,3) apresentando valores maiores que os de maior tempo de escolarização. No grupo com doenças de risco, participantes com hipertensão arterial sistêmica (47,9) e doenças que afetam o sistema imunológico (47,8) registraram valores maiores, enquanto os com doenças autoimunes tiveram valores menores (22,5) que os demais grupos (F = 10,79; p < 0,001; η ^2^ = 0,022).

A dimensão benefícios percebidos foi significante para os diferentes participantes com base no sistema de saúde utilizado (F = 4,32; p = 0,037; η ^2^ = 0,007), uma vez que os participantes usuários de sistema privado de saúde (21,5) apresentaram valores mais altos do que os usuários do sistema público (18,2).

A última dimensão do modelo que apresentou diferenças significantes entre participantes foi barreiras percebidas (F = 3,79; p = 0,004; η ^2^ = 0,014), na qual usuários de transporte público apresentaram valores menores que os demais grupos de transporte. Não encontramos em nenhuma dimensão diferenças significativas com relação ao sexo dos participantes, nem correlação significativa entre a idade e as respostas das escalas.

### Perfil Individual

O uso da escala centiMax ^®^ permite uma análise gráfica de cada um dos participantes, de forma direta e rápida. Apresentamos na Figura 2 o perfil de dois participantes que apresentam valores de soma dos itens da escala praticamente iguais (participante A – 45,9; participante B – 45,7). No entanto, claramente podemos identificar diferentes crenças sobre o covid-19. A linha vertical no valor de 50 unidades da escala representa a percepção alta do item em questão. De uma perspectiva de aplicação, a Figura 2 mostra como nosso questionário associado ao uso da escala de razão nos dá uma gradação quantitativa e refinada, permitindo ao participante expressar suas percepções e intensidades respectivas com maior nível de detalhamento quando comparado com outras escalas ordinais ou nominais.

## DISCUSSÃO

Nosso trabalho cumpre com sucesso nosso objetivo de mapear quantitativamente a percepção sobre os comportamentos de risco diante do risco de contágio por coronavírus utilizando o modelo de crença em saúde associado a uma escala psicofísica de ancoragem verbal, possibilitando uma representação detalhada das percepções individuais. Essa abordagem se mostrou muito eficiente, uma vez que é capaz de identificar em participantes com o mesmo valor de percepção de risco perfis muito diferentes nas variáveis que contribuem para essa percepção.

Os resultados obtidos identificaram que alguns fatores são de significante importância para o entendimento da percepção de risco. O tipo de transporte utilizado para se locomover diariamente afeta significativamente a percepção de risco, com os usuários de transporte público percebendo uma maior susceptibilidade à contaminação que os usuários de veículo próprio e os que caminham para suas atividades. A mesma percepção de uma maior susceptibilidade foi encontrada em pessoas com renda muito baixa e indivíduos que apresentam principalmente doenças autoimunes e doenças que afetam o sistema imunológico (alergias e reumatismos, por exemplo).

Esses achados são importantes fontes de informação para adotarmos políticas públicas que busquem a maior abrangência possível. É positiva a percepção de que o uso de transporte público apresenta um maior risco de contrair covid-19 do que o transporte privado próprio. No entanto, o uso de veículos privados coletivos, como os transportes por aplicativos, apresentaram uma variabilidade grande e, portanto, não diferiram de nenhum dos grupos. Assim, entendemos que esse é um público-alvo interessante para o aumento de informações sobre os riscos de contágio. A baixa renda também foi um fator importante, que deve ser encarado de forma complexa, uma vez que está associado à quantidade e qualidade reduzidas da informação disseminada, condições de moradia que favorecem a contaminação e dificuldade de interromper as atividades diárias por questões econômicas.

Chamou-nos a atenção o fato de que pessoas com doenças que estão sendo associadas ao maior risco de contágio por covid-19, como diabetes mellitus e hipertensão arterial sistêmica ^1,3^ , não apresentam percepção de susceptibilidade para o risco de contaminação com diferença significante em relação ao grupo de pessoas sem autorrelato de doenças crônicas. Uma possível explicação pode se dar pelo fato de que estes pacientes são, em sua grande maioria, assintomáticos e permanecem com a doença estável e controlada clinicamente, levando-os a se comportar como indivíduos sem doenças crônicas. Esse resultado permite que desenvolvamos políticas de disseminação de informação com maior ênfase ou até mesmo veiculações específicas para este grupo de risco.

Com relação à intensidade de sintomas e característica de evolução da doença, os participantes com níveis de escolaridade mais baixos de nossa amostra (secundário incompleto e completo) demonstraram maior preocupação com os possíveis sintomas, pois suas representações da severidade percebida foram maiores do que os demais níveis de escolaridade. A percepção de maior severidade dos sintomas pode levar essas pessoas a procurarem os serviços de saúde mais precocemente. Esse é um problema que está sendo frequentemente discutido, pelo risco de exposição desnecessária ao covid-19 e a outras doenças graves na ida a uma instituição de saúde sem a real necessidade.

Outro resultado curioso que obtivemos para a percepção de severidade foi em relação às doenças crônicas. Participantes com hipertensão arterial sistêmica e doenças que afetam o sistema imunológico registraram maior susceptibilidade que os demais grupos. Entretanto, os participantes com doenças autoimunes registraram valores significativamente menores que os demais, sugerindo que as precauções que tomam para o tratamento e cuidado de suas doenças crônicas estão sendo positivamente percebidas como fatores protetivos à exposição. O tipo de sistema de saúde utilizado teve impacto na percepção de benefícios quanto ao acesso e tratamento em caso de covid-19, com os usuários do sistema público se percebendo menos beneficiados em relação aos participantes que utilizam sistemas privados de saúde.

Os resultados obtidos nos perfis individuais possibilitam análises quantitativas diretas, que são altamente informativas. Por exemplo, se observarmos a questão 1 do item motivação à saúde na Figura 2, vemos que o participante A acredita 30 unidades que o comportamento de lavar as mãos previne o contágio pelo vírus. Já o participante B acredita 120 unidades que esse comportamento é benéfico. Por se tratar de uma escala de razão, podemos diretamente afirmar que o participante B acredita quatro vezes mais que o participante A na eficácia de lavar as mãos como hábito saudável de comportamento que deve ser incentivado ou aumentado. Quando comparamos a questão 2 do item severidade percebida, o participante A acredita duas vezes mais na chance de ter sintomas e complicações graves que o participante B. Se tivéssemos utilizado uma escala tipo Likert de 5 pontos, teríamos no primeiro caso o resultado muito alto (ou valor 5, se o escore fosse numérico) para o participante B e baixo (valor 2) para o participante A. No segundo caso, teríamos para o participante A o resultado moderado (ou valor 3) e para o participante B o resultado baixo (valor 2). Nesse exemplo fica nítida a diferença entre o uso de uma escala de razão contra uma escala ordinal, cuja sensibilidade e resolução prejudicam a real identificação das representações dos participantes.

**Figura 2:** Perfil de representação sobre a crença de contágio por coronavírus (causador da covid-19) para classificação em unidades da escala centiMax ® (Cmax) da intensidade da percepção do risco para duas pessoas com os escores por soma muito semelhantes (A = 45,7 e B = 45,9 unidades; chance de risco autodeclarada como alta). A linha vertical a 50 unidades indica uma percepção de risco de contágio “alta” na escala (vide a Figura 1).

É também de importância significativa falarmos sobre a metodologia utilizada neste estudo. Embora o modelo de crença em saúde tenha sido desenvolvido há mais de 60 anos ^8,^ ^9^ e ter aplicação em diversas áreas da saúde – como educação em oftalmologia ^29^ , estudo de aspectos comportamentais na psiquiatria do comportamento alimentar ^6^ , uso e abuso de drogas ilícitas injetáveis ^30^ , clínica médica endocrinológica da diabetes mellitus ^12^ , entre outras – o uso habitual desse modelo inclui questionários abertos ou semiestruturados, utilizando escalas do tipo Likert para a realização de medidas das dimensões psicológicas. Nosso estudo é um claro avanço no uso desse modelo, uma vez que aplica uma escala de razão com ancoragem verbal, permitindo assim medidas de razão e, portanto, sendo de alto poder de detalhamento de informação e de forma quantitativa. Esse ponto metodológico merece atenção, pois um bom questionário pode ter seu poder de captura de informação muito prejudicado se uma métrica inadequada ou de baixa capacidade informativa for utilizada. Os trabalhos de S.S. Stevens em medidas psicológicas e escalonamento psicofísico ^21,23^ expandiram nossos entendimentos sobre as possibilidades métricas aplicadas ao universo psicológico. As escalas do tipo Likert são de origem ordinal e, portanto, de métrica posicional, e seus descritores indicam apenas ordens, sem condições de designar a distância entre elas. Por isso, escalas ordinais utilizam medidas estatísticas não paramétricas como moda, frequência, associação e correlação categórica. Calcular médias em escalas do tipo Likert, mesmo que a ordem seja representada numericamente, é um erro fundamental de medida. Por outro lado, medidas em universos de razão, por apresentarem origem absoluta de contínuos matemáticos, permitem estatísticas da mais alta ordem. Nosso trabalho, portanto, apresenta uma métrica de altíssima qualidade informativa e objetividade métrica.

Nosso grupo já tem experiência na construção de medidas e escalas psicofísicas de ordem intervalar e de razão para estudos de diversos *continua* psicológicos, como sobre o perfil de sintomas em pacientes com depressão maior ^19^ , atitudes sexuais ^31^ e conceito de cor ^32^ . Estimulamos o uso desses modelos de escalonamento psicofísico, em especial as escalas de ancoragem verbal, porque são de fácil aplicação e entendimento da tarefa a ser realizada, além da vantagem de estarem associados a medidas quantitativas de grande poder de informação, intervalares e de razão. Esses fatores geram um grande potencial para estudos mais detalhados e quantitativos em saúde pública, a qual, assim como a psicologia, encara muito frequentemente características complexas do comportamento humano em relação a questões de prevenção e risco de saúde e adoecimento.

Nosso trabalho apresenta algumas limitações evidentes, que podem ter algum impacto nos resultados obtidos. Nossa amostra apresenta um adensamento em populações com renda moderada a alta, o que pode afetar a representatividade desta população. Portanto, estudos posteriores deverão buscar corrigir esta não homogeneidade social da amostra.

Como conclusão, nosso questionário baseado no modelo de crença em saúde associado a uma escala de razão com ancoragem verbal é um importante instrumento para o entendimento da percepção populacional a respeito dos riscos de contágio por coronavírus de maneira quantitativa e, portanto, muito mais detalhada e informativa que outros modelos de questionários e métricas psicológicas aplicados habitualmente, como as escalas ordinais tipo Likert. A análise gráfica permite um acesso rápido do perfil individual, possibilitando o desenvolvimento de estratégias de informação e abordagens mais individualizadas, o que certamente terá um impacto maior na eficiência da comunicação.
